# Community-based cultural heritage preservation through the *Purok* system: a mixed-methods case study in a Philippine municipality

**DOI:** 10.3389/fsoc.2026.1813600

**Published:** 2026-06-16

**Authors:** Emily Costan

**Affiliations:** College of Education, Arts and Sciences, Cebu Technological University Danao Campus, Danao, Cebu, Philippines

**Keywords:** community participation, cultural heritage preservation, mixed-methods design, Purok system, sustainable development

## Abstract

The preservation of cultural heritage is a universal concern that has to reach the local community to protect its history, identity, and pride. But despite various national and local heritage initiatives in the Philippines, limited research has examined how micro-level units function, such as the Purok system. This study employed a sequential explanatory mixed-methods design to examine the cultural heritage preservation of the Municipality of Liloan, Cebu. A survey of 264 members from six puroks was analyzed using linear regression models, and a focus group discussion with 20 purposively selected participants was conducted. The quantitative results show that awareness plays a more critical role than participation in influencing local government initiatives, indicating a positive relationship with the independent variables. This implies that higher awareness and greater participation are generally associated with more substantial government-led preservation efforts. Further, the participation in cultural activities has a positive, statistically significant effect on community initiatives; however, awareness of cultural heritage preservation shows a negative relationship, suggesting that awareness alone may not translate into active community preservation initiatives. The qualitative analysis emphasizes themes of lived engagement, stewardship, and motivational pathways, demonstrating that preservation is reinforced through shared responsibilities, social interaction, collective identity, continuity, and community meaning rather than solely through knowledge. The findings also reveal that younger residents show lower levels of engagement, yet their digital skills offer opportunities for innovative documentation and promotion of heritage assets. These results indicate the potential of the Purok system as a community-based framework for strengthening participatory and sustainable cultural heritage preservation efforts.

## 1 Introduction

In many societies around the world, culture serves as a foundation of collective identity, social cohesion, and the transmission of shared meanings across generations. Culture is a core part of how individuals and communities perceive themselves, their history, language, values, and rituals (Hiswara et al., 2023). In this sense, culture is expressed through tangible and intangible forms that communities seek to sustain across generations as heritage. Heritage, as an aspect of culture, is acquired from the past and encompasses both tangible objects and intangible skills and knowledge. Heritage concerns our history, present, and future (Gilmour, 2007). Places, cultures, customs, values, artifacts, and physical structures are all part of heritage. The potential of both tangible and intangible forms of legacy to support sustainability has become increasingly recognized over the past few decades. Under the Sustainable Development Goals (SDGs) on Sustainable Cities and Communities, heritage is recognized as an essential part of safeguarding cultural and natural legacy. Beyond restoring buildings, heritage conservation can contribute to many of the 17 SDGs. However, current frameworks often fail to connect these goals with practical, on-the-ground actions (Naheed and Shooshtarian, 2022). It also provides a foundation for advancing cultural heritage preservation at the community level.

Cultural heritage preservation involves the active protection and transmission of tangible and intangible traditions, practices, and artifacts across generations. Cultural heritage consists of physical objects, living traditions, and natural heritage (Blake, 2000). Works of art with enduring worth and that should be preserved for future generations, such as monuments, historic sites, and relics, are considered tangible cultural heritage. These are tangible relics that were produced, maintained, or abandoned by earlier societies. Intangible cultural heritage comprises non-material components that communities and individuals perceive as part of their cultural heritage, such as practices, skills, and expressions, along with the items and cultural spaces that accompany them (Banda et al., 2024). Cultural heritage is commonly transmitted orally, visually, and through traditions and customs across generations. Preserving it ensures that this knowledge will not be lost and can be passed on to future generations. Cultural preservation ensures the transmission of traditional knowledge, skills, languages, crafts, artifacts, and ceremonies to future generations. In the absence of this continuity, knowledge gaps emerge, and cultural expressions may become extinct (Ye et al., 2025). These challenges highlight the importance of examining how cultural preservation initiatives are implemented in the Philippines to sustain heritage and community practices.

Cultural heritage preservation in the Philippines involves initiatives that raise community awareness and engagement, ensuring that local traditions, practices, and historical knowledge are actively maintained and transmitted (Kintanar and Barretto-Tesoro, 2020). National, regional, and local agencies develop policies and guidelines to protect cultural heritage, while heritage societies, cultural associations, and community projects support preservation through events, campaigns, and resource mobilization. Preserving local cultural heritage is essential for safeguarding community identity, history, and traditions, serving as a repository of collective memory that reflects past experiences and values while guiding future generations in understanding their cultural roots (Pai et al., 2025). Losing cultural heritage equates to losing parts of identity, disrupting continuity, and weakening community ties (Hiswara et al., 2023). Local culture preservation involves various agents and stakeholders who play crucial roles in protecting and promoting cultural heritage. Among these, the academe contributes to cultural heritage preservation through education, documentation, and research. They may offer programs in cultural studies, anthropology, archeology, and heritage management, providing students with the knowledge and skills to contribute to cultural preservation efforts. It is essential to foster local cultural awareness among young learners, as it is more feasible, beneficial, achievable, and realistic (Mteti et al., 2025). One such smaller local community unit is the *Purok* system, which functions within a barangay and organizes residents around local initiatives.

The *Purok* system, a smaller administrative unit within a barangay in the Philippines (Matthies, 2017), plays a crucial role in preserving cultural heritage by enabling residents to identify, document, and safeguard traditions within their neighborhoods. Functioning similarly to a barangay, *Puroks* have organized committees and appointed chairpersons who link community members with local government units, facilitating direct participation in regional initiatives (De Leon, 2021). They have the potential to serve as hubs for cultural exchange, intergenerational knowledge transfer, and community-led preservation efforts, including restoration, awareness campaigns, and collaborations with heritage organizations. This suggests an opportunity to explore how the system can contribute to the sustainable preservation and promotion of cultural heritage for future generations. The role of the community is the dominant factor in the effectiveness of heritage preservation efforts (Rami et al., 2023). While most research has focused on the *Purok* system in the context of disaster resilience, its role in cultural heritage preservation remains unexplored, a system dynamic that is not fully understood. To uncover these dynamics, this study employed a sequential explanatory mixed-methods design (Creswell and Clark, 2017) that integrated quantitative survey data with qualitative thematic analysis.

The remainder of the paper is organized as follows. Section 2 presents the literature review. Section 3 describes the methods and details the mixed-methods approach used in the study. Section 4 presents the qualitative and quantitative results in an integrated manner. Section 5 discusses the implications of the integrated findings. Section 6 presents the conclusions, acknowledges the limitations, and outlines directions for future research.

## 2 Literature review

This section lays out the theoretical and conceptual bases for understanding the link between heritage conservation and grassroots governance. It synthesizes various scholarly views, beginning with Theoretical Perspectives on Cultural Heritage Preservation, which look beyond mere monument preservation to consider how social value and cultural capital are shaped and preserved. Then, it explores The Role of the *Purok* System in Local Community Dynamics, analyzing how this Filipino sub-village structure functions as a key platform for civic involvement and local governance. This literature offers two perspectives: one on the significance and purpose of heritage, and the other on the processes of community-led action.

### 2.1 Theoretical perspectives on cultural heritage preservation

Cultural heritage preservation is grounded in various theoretical perspectives that explain how heritage is valued, safeguarded, and passed down through generations. These perspectives see heritage not just as tangible objects but also as living practices influenced by social, cultural, and political factors. The recognition of heritage as a social construct highlights that what communities choose to preserve reflects shared meanings, identities, and cultural values rather than fixed, objective criteria. Preservation is increasingly seen not simply as physical conservation but as an active, dynamic process of sustaining cultural identity and continuity through community practices and collective memory. The Cultural Capital Theory views heritage as a resource that strengthens identity, social bonds, and intergenerational continuity (Bourdieu, 2018). In this context, capital represents accumulated labor that, when utilized, gives individuals or groups social leverage. The theory broadens the concept of capital beyond economic assets, incorporating cultural and social dimensions. This perspective enables a more detailed analysis of how power, privilege, and social layers are gained, passed on, and maintained within society, revealing the complex strategies people and groups use to navigate the social world. This leads to a hierarchy where elite narratives are preserved while the heritage of marginalized groups is overlooked, making preservation a tool for both social cohesion and social distinction.

Additionally, cultural heritage is a social process, not a fixed attribute of objects, with meanings mediated by institutions and communities (Smith, 2006). Preservation reflects power and cultural priorities, not just history. Community participation is vital, shaping how heritage is defined and valued locally. Shifted from a purely material focus to a more human-centric approach, Values-Based Heritage Management asserts that a site’s significance is determined by the “values” attributed to it by various stakeholders. These values are typically categorized into esthetic, historic, scientific, social, or spiritual dimensions. Unlike traditional models that focus primarily on a building’s physical structure, this approach recognizes that values are subjective and evolve (Spennemann, 2011). Management within this framework requires a careful balance, a dialog between experts and local communities to ensure preservation efforts reflect what people currently find meaningful, rather than only the physical elements.

Among the perspectives discussed, Cultural Capital Theory (Bourdieu, 2018) serves as the primary theoretical framework of this study. To support analytic triangulation, the Uses of Heritage framework was also employed, particularly through its socially constructed and interpretive perspective (Smith, 2006). The theory underpins the assumption that cultural heritage preservation is sustained through community participation, shared practices, and the intergenerational transmission of cultural knowledge. Recent heritage studies further reinforce this view, highlighting the importance of “living heritage,” community participation, and social value in sustaining cultural continuity and well-being. In the context of conservation science, the inclusion of Indigenous and local knowledge, along with the strengthening of participatory and community-based approaches in conservation governance, is recognized as essential for achieving more equitable and context-responsive heritage and conservation practices (Maliao and Tóthmérész, 2026). Taken together, these frameworks, along with recent applications, underpin the assumption that cultural heritage preservation is sustained through community participation, shared practices, and the intergenerational transmission of cultural knowledge within socially embedded systems such as the Purok.

### 2.2 The role of the Purok system in local community dynamics

In the Philippines, the *Purok* system is an established community-based structure that divides villages into smaller, manageable units with local coordinators, facilitating direct engagement with residents (De Leon, 2021). It operates at the micro level of local governance within the barangay, the smallest administrative unit of the government system (Matthies, 2017). It is promoted as a voluntary, self-organized sub-village-level initiative. Research indicates that these units are essential for improving service delivery and community involvement. It bridges the gap between the formal local government and residents, with a shared responsibility (Lapiz, 2024) by dividing villages into smaller, independently governed units (De Leon, 2021). When organizing households into smaller clusters, it became more effective to implement the program through local leaders, including health programs, disaster risk reduction strategies, and solid waste management initiatives. Through this system, Barangay Health Workers (BHWs) and *Purok* leaders can monitor detailed census data on vulnerable populations, including infants, the elderly, and pregnant women (Dayrit et al., 2018). Where the system guides households to engage in community-based risk management, active health committees exhibit noticeably higher compliance with national health mandates than those without a structured sub-unit system (Matthies, 2017).

The system also clearly distinguishes itself from traditional community-based disaster risk management methods and holds considerable promise for enhancing resilience in disaster-prone areas (Matthies, 2017). They operate at the community level, enabling local leaders to disseminate early warnings, mobilize resources quickly, and initiate evacuations even before municipal responders arrive (Matthies, 2017). This localized approach enables the development of Purok-specific hazard maps that identify households most at risk of flooding or landslides, ensuring no one is missed during emergencies. Residents are empowered to serve as first responders when the *Purok* system is integrated into the Barangay Disaster Risk Reduction and Management (BDRRM) framework, thereby bridging the gap between community survival and national policies. Furthermore, maintaining community hygiene and preventing disease depend heavily on solid waste management (SWM). Research shows that *Purok*-level “Materials Recovery Facilities” (MRFs) and small campaigns are more sustainable due to social pressure and shared responsibility within neighborhoods (Matthies, 2017). These micro-units help local governments improve source segregation of waste, aligning with national standards (Lapiz, 2024). This idea is also supported by theoretical applications of participatory governance, particularly emphasizing the role of youth as the most engaged members of society, whose perspectives may shape cultural heritage in distinct ways (Del Baldo and Demartini, 2021). Focusing health and sanitation efforts at the *Purok* level encourages residents to actively maintain a clean, disease-free environment, shifting from service recipients to participants (Brillantes and Fernandez, 2011).

Beyond administrative efficiency, the *Purok* system significantly influences social cohesion and peace-building within the neighborhood. The informal yet structured nature of the *Purok* allows for rapid conflict resolution through the “Katarungang Pambarangay” (Barangay Justice System) before disputes escalate to higher legal levels (Brillantes and Fernandez, 2011). Also, the system encourages “Bayanihan” or communal unity, as residents are more likely to participate in local projects when they feel a direct connection to their immediate neighbors. This localized approach ensures that even the most marginalized sectors have a direct line to communal resources and decision-making processes (De Leon, 2021). Thus, the *Purok* system functions not only as a geographical boundary but also as a dynamic social engine that drives grassroots development and local security.

### 2.3 Integration of cultural heritage and Purok-based heritage dynamics

This study argues that heritage preservation at the community level is best understood as the interaction between cultural capital and micro-level governance structures, particularly the purok system. The theoretical explanation for why people and communities value and participate in heritage practices in this framework is cultural capital. Rather, the purok system provides the structural context through which such engagement is socially organized and enacted. In particular, embodied cultural capital, such as local knowledge, historical consciousness, and cultural dispositions, determines individuals’ willingness to engage in activities to preserve heritage. The purok system provides structured opportunities for participation, collective action, and intergenerational transmission, thereby facilitating the social conditions for activating this capital. The preservation of heritage is not only dependent on formal institutions but is heavily influenced by localized social structures that enable the mobilization of cultural capital into collective practice. Concrete measures of cultural capital and community participation in the purok system can reveal empirical differences in heritage preservation behavior among individuals.

## 3 Methods

This section describes the research design, participants, data collection procedures, and data analysis techniques employed to investigate cultural heritage preservation initiatives. Both quantitative and qualitative approaches are detailed, including the rationale for using mixed-methods design and the procedures for integrating findings.

### 3.1 Research design

This study employed a sequential explanatory mixed-methods research design (Creswell and Clark, 2017) to examine the cultural heritage preservation program of the Municipality of Liloan, Cebu, Philippines, implemented through the *Purok* system. Quantitative data were first collected and analyzed to identify patterns in cultural heritage preservation awareness and participation, followed by qualitative data to provide deeper insights into participants’ experiences and perspectives. Quantitative and qualitative findings were integrated in the discussion section to provide a comprehensive understanding of the influence of community engagement and awareness on local government preservation initiatives.

### 3.2 Background of the case municipality

The study was conducted in the Municipality of Liloan, Cebu, Philippines, a first-class coastal municipality in northern Cebu known locally as “the Light of the North” and composed of 14 barangays with over 230 *Puroks* organized for community governance and development (Municipality of Liloan, 2026). The *Purok* structure controls existing social ties, trust, and local knowledge, allowing initiatives to be contextualized to the culture and practices. Liloan’s *Purok* system has been institutionalized to promote grassroots participation in local programs, including environmental protection, clean-and-green initiatives, and community performance evaluation, with *Puroks* regularly recognized for their contributions (Palaubsanon, 2017). Because of enabled close coordination, localized participation, and bottom-up organization, they are well-suited for documenting and supporting cultural heritage preservation initiatives, where community awareness, participation, and shared stewardship are critical.

### 3.3 Participants and sampling

The study employed separate sampling designs for the quantitative and qualitative components. For the quantitative component, a two-stage sampling approach was used. At the first stage, two barangays were randomly selected from a total of 14 barangays in the Municipality of Liloan, Cebu, Philippines. At the second stage, *Puroks* within the selected barangays served as the sampling units, and six *Puroks* (10% of the total 60 across the selected barangays) were randomly selected. At the respondent level, an initial sample of 64 household representatives was obtained through purposive nomination by purok presidents based on active involvement in community activities and familiarity with cultural heritage preservation practices. To address concerns about representativeness and potential selection bias, a second wave of data collection was conducted, during which nomination procedures were removed and additional respondents were recruited through voluntary participation, yielding an additional 200 respondents. There are 264 total survey respondents.

For the qualitative component, the first phase involved a focus group discussion (FGD) with an initial group of 10 participants purposively selected from survey respondents based on their level of engagement in cultural activities and their ability to articulate experiences related to cultural heritage preservation. Following preliminary analysis of the FGD transcripts, Purok members with lower levels of involvement or non-participation in cultural heritage programs were identified, particularly among youth respondents. To capture a broader range of perspectives and address this gap, additional focus group discussions (FGDs) were conducted with 10 participants drawn from these identified clusters. This resulted in 20 FGD participants, categorized into Group 1: Actively Engaged Informants and Group 2: Less-Involved Informants. Table 1 presents the demographic profile of the survey respondents and FGD informants.

**Table 1:** Demographic profile of the survey respondents and FGD informants.

Survey respondents (*N* = 264)		*n*	%
Sex	Male	85	32.20%
	Female	179	67.80%
Age	18 to 30 years old	34	12.88%
	31 to 40 years old	65	24.62%
	41 to 50 years old	74	28.03%
	51 to 60 years old	41	15.53%
	61 years old and above	50	18.94%
*Purok* role classification	Member	225	85.23%
	Officer	32	12.12%
	Adviser	7	2.65%

FGD informants (*N* = 20)		*n*	%
Sex	Male	6	30.00%
	Female	14	70.00%
Age	18 to 30 years old	10	50.00%
	31 to 60 years old	5	25.00%
	61 years old and above	5	25.00%
*Purok* role classification	Member	7	35.00%
	Officer	12	60.00%
	Adviser	1	5.00%

### 3.4 Instrument

A structured survey questionnaire was used to examine the Local Cultural Heritage Preservation Program. The instrument consisted of two parts: the first collected demographic information, and the second measured latent constructs related to cultural heritage preservation using validated indicators. Cultural Heritage Preservation Awareness was measured using 11 items, while Participation in Cultural Activities was measured using 9 items. Preservation strategies were assessed through two constructs: Local Government Units (LGU) Cultural Heritage Preservation Strategies (6 items) and Community Cultural Heritage Preservation Initiatives (9 items). All items were adapted from established instruments developed by Hiswara et al. (2023), Jagodzinska (2023), and Ariffin et al. (2023), with minor contextual modifications to ensure suitability to the local setting while preserving the original construct dimensions. Given these contextual modifications, content validation was conducted by three subject matter experts to ensure appropriateness and construct validity before data collection. The validation process consisted of three iterative rounds until consensus was reached on the final instrument. Following validation, reliability analysis demonstrated strong internal consistency, with Cronbach’s alpha coefficients ranging from 0.860 to 0.940 as reported in Table 2.

**Table 2:** Zero-order correlations of the study variables.

Study variables	1	2	3	4
Cultural heritage preservation awareness	1			
Participation in cultural activities	0.552**	1		
LGU initiatives	0.462**	0.483**	1	
Community initiatives	0.186**	0.580**	0.488**	1
Mean	3.594	3.527	3.506	3.406
Standard deviation	0.276	0.416	0.404	0.433
Cronbach’s alpha	0.791	0.883	0.767	0.862

**Correlation is significant at the 0.01 level (2-tailed).

Based on the quantitative results, an interview guide was developed to further examine and clarify the statistical findings. The guide underwent language validation to ensure clarity and cultural appropriateness and was subsequently validated by two field experts holding doctoral degrees (PhD in Education, major in Research and Evaluation). As part of the inclusion criteria, validators had established publication records in peer-reviewed journals within their respective fields. The interview guide was translated into the local dialect for clarity, and the interview transcripts were translated into English and reviewed by a language expert to ensure accuracy and consistency of meaning.

### 3.5 Data analysis

This section presents the methods and procedures used to analyze both quantitative and qualitative data collected for the study.

#### 3.5.1 Quantitative data analysis

**Figure 1:**
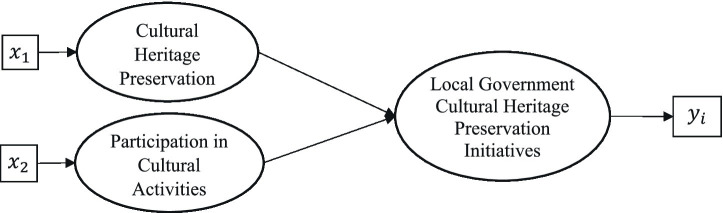
The proposed regression model 1.

**Figure 2:**
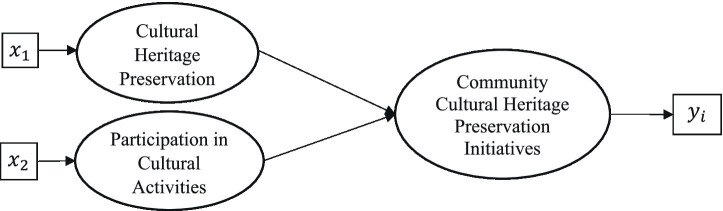
The proposed regression model 2.

Quantitative data were analyzed using means and standard deviations to summarize the respondents’ demographic profiles and their perceptions of cultural heritage preservation awareness, participation, and strategies. Zero-order correlations were computed to examine relationships among the key variables, and regression analyses were conducted to assess the influence of Cultural Heritage Preservation Awareness and Participation in Cultural Activities on LGU and Community Preservation Initiatives. The multivariate data analysis allowed examination of the relationships among variables and how awareness and participation in cultural activities affected local government and community preservation efforts. The proposed regression models were presented in Figures 1, 2.

The regression models are grounded in Bourdieu’s (2018) Cultural Capital Theory, which views cultural knowledge, skills, and participation in cultural practices as forms of capital that influence social actions and institutional outcomes. In this context, high levels of cultural heritage preservation awareness and participation in cultural activities reflect substantial cultural capital, which can lead to stronger support for Local Government Cultural Heritage Preservation Initiatives.

#### 3.5.2 Qualitative data analysis

Qualitative data from the focus group discussion (FGD) were audio-recorded, transcribed verbatim, and translated into English, with the translation validated by a language expert to ensure accuracy. The transcribed data were organized in NVivo Pro, which facilitated word-frequency analysis, source tracking, and the initial classification of codes into nodes (Jorolan et al., 2025). The qualitative data were analyzed using a concept-driven thematic analysis informed by Smith (2006). Initial open coding identified meaningful units from the transcripts, which were then organized using six guiding categories: heritage as a cultural process, heritage as experience, heritage as identity, the intangibility of heritage, memory and remembering, and heritage as performance. Codes were iteratively refined and clustered into broader themes grounded in the data and guided by this theoretical lens.

This analytic process was further interpreted through Cultural Capital Theory (Bourdieu, 2018), which situates cultural knowledge, practices, and participation as forms of capital transmitted and reproduced within communities. The resulting themes were therefore examined in relation to how heritage awareness, participation, and intergenerational engagement function as mechanisms for the accumulation and transfer of cultural capital within the *Purok* system.

### 3.6 Ethical review clearance

The study was conducted with an approved institutional ethics review protocol from the Local Ethics Review Committee (LREC). The ethics review certificate was dated November 20, 2024, and the data were collected from August 2025 to December 2025. The study was classified as ‘Exempted’ because it posed a low risk, and strict measures were applied to protect participants, including obtaining informed consent, maintaining confidentiality and anonymity, ensuring voluntary participation, and ensuring the safe handling of data. Participants were given clear information about the purpose, procedures, potential risks and benefits, and privacy measures. Ethical standards were followed throughout data collection, analysis, reporting, and publication.

## 4 Results

This section presents the study’s findings, organized into quantitative and qualitative analyses. The quantitative results provide numerical evidence on the relationships among cultural heritage preservation awareness, participation in cultural activities, and preservation strategies. In contrast, the qualitative results offer contextual explanations and deeper insights from informants’ perspectives.

### 4.1 Quantitative results

The quantitative results include overall descriptive measures, comparisons among the study variables, and multivariate regression analyses. Descriptive statistics show levels of awareness, participation, and perceptions of preservation strategies, while regression analyses examine the predictive relationships between the explanatory variables and the outcomes. The zero-order correlations, descriptive measures, and the reliability analysis of the study variables are presented in Table 2.

Following the interpretation guidelines, correlation coefficients around 0.10 are considered weak, around 0.30 moderate, and 0.50 or higher strong (Gignac and Szodorai, 2016). Based on these criteria, cultural heritage preservation awareness demonstrated a strong positive relationship with participation in cultural activities (*r* = 0.552, *p* < 0.01) and a moderate positive relationship with LGU initiatives (*r* = 0.462, *p* < 0.01). However, its relationship with community initiatives was weak, though still statistically significant (*r* = 0.186, *p* < 0.01). participation in cultural activities was strongly associated with community initiatives (*r* = 0.580, *p* < 0.01) and moderately associated with LGU initiatives (*r* = 0.483, *p* < 0.01). Likewise, LGU initiatives and community initiatives exhibited a moderate positive relationship (*r* = 0.488, *p* < 0.01). These findings suggest that both institutional and community-driven efforts are linked to greater engagement in cultural activities and awareness of cultural heritage preservation.

The descriptive statistics indicate that respondents generally reported moderately high levels across all study variables, with cultural heritage preservation awareness obtaining the highest mean (*M* = 3.594, SD = 0.276), followed by participation in cultural activities (*M* = 3.527, SD = 0.416), LGU initiatives (*M* = 3.506, SD = 0.404), and community initiatives (*M* = 3.406, SD = 0.433). Reliability analysis further showed acceptable to high internal consistency, with Cronbach’s alpha values ranging from 0.767 to 0.883, indicating that the measurement scales used in the study were reliable.

**Figure 3:**
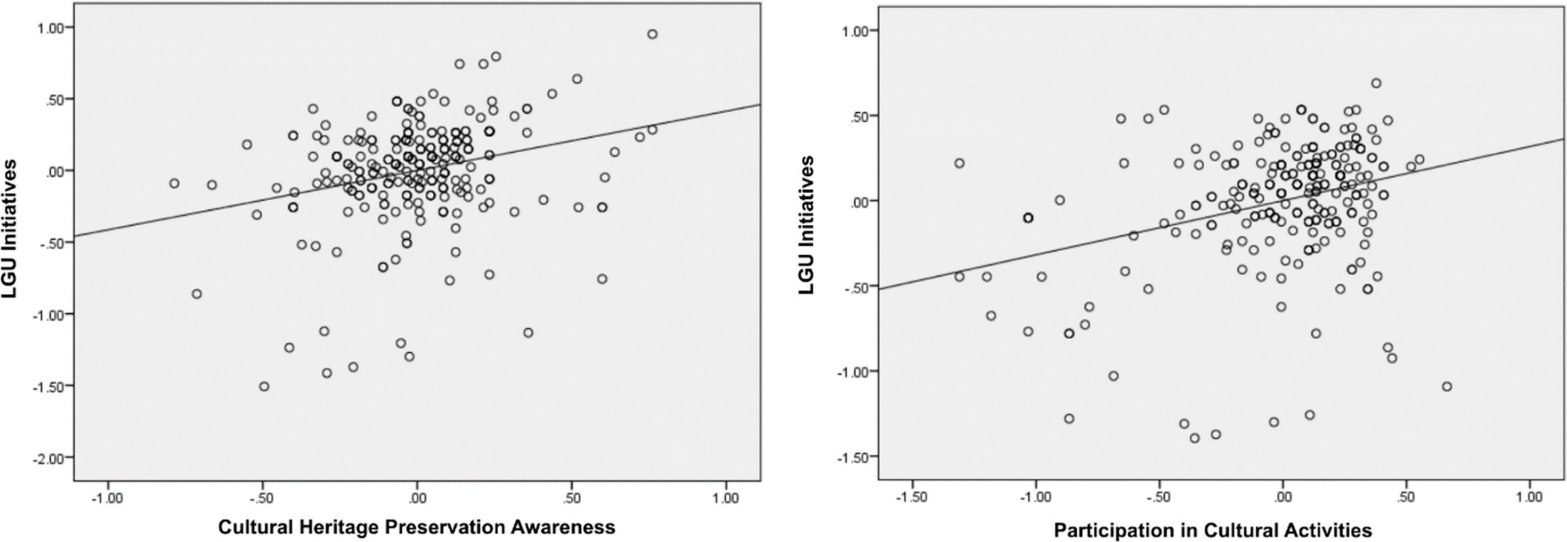
Comparative scatter plots for the regression model presented in Table 3.

Based on the regression results presented in Table 3, the regression equation can be written using the unstandardized coefficients ( β ) as follows:

$$Y=0.897+0.414X_{1}+0.318X_{2}$$ (1)

where Y is the local government cultural heritage preservation initiatives, $X_{1}$ is the cultural heritage preservation awareness, and $X_{2}$ is the participation in cultural activities. Equation 1 suggests that awareness of cultural heritage preservation and participation in cultural activities predict local government unit initiatives. Cultural heritage preservation awareness has a positive, significant effect, indicating that greater awareness is associated with stronger perceptions of government-led preservation efforts. Participation in cultural activities also has a positive impact, but it is only marginally significant, suggesting that active engagement contributes to government initiatives, though the effect is weaker. Results with p -values slightly above the conventional 0.05 threshold (e.g., p ≈ 0.05–0.10) are commonly described in the literature as marginally significant or approaching significance, indicating weak evidence against the null hypothesis rather than definitive statistical support (Pritschet et al., 2016). These findings suggest that awareness plays a more critical role than participation in influencing local government unit preservation strategies. The scatter plots (see Figure 3) show a positive relationship between the independent variables and local government unit initiatives, indicating that higher awareness and greater participation are generally associated with more substantial government-led preservation efforts.

**Table 3:** Regression coefficients predicting local government cultural heritage preservation initiatives.

Predictor	*B*	(*β*)	*t*-statistics	*p*-value	VIF
Constant	0.897		3.217	0.001	
Cultural heritage preservation awareness	0.414	0.282	4.490	<0.001	1.438
Participation in cultural activities	0.318	0.327	5.211	<0.001	1.438

*B*, unstandardized coefficient; β , standardized coefficient; VIF, variance inflation factor.

**Figure 4:**
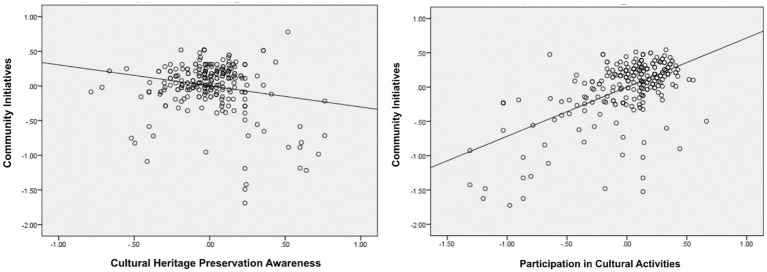
Comparative scatter plots for regression model presented in Table 4.

Table 4 presents a regression analysis predicting community cultural heritage preservation initiatives, with cultural heritage preservation awareness and participation in cultural activities as predictor variables. The findings indicate that the regression model identified both predictors as statistically significant contributors to community cultural heritage preservation initiatives. The regression results indicate that participation in cultural activities has a positive and statistically significant effect on community initiatives, suggesting that active engagement is an essential predictor of community-led cultural heritage preservation efforts. In contrast, cultural heritage preservation awareness shows a statistically negative relationship, suggesting that awareness alone may not translate into active community preservation initiatives when participation in cultural activities is also considered in the model (see Figure 4). It may indicate that practical involvement in cultural activities plays a more influential role than awareness itself in predicting community-based preservation efforts.

**Table 4:** Regression coefficients predicting community cultural heritage preservation initiatives.

Predictor	*B*	(*β*)	*t*-statistics	*p*-value	VIF
Constant	1.971		6.960	0.001	
Cultural heritage preservation awareness	−0.304	−0.193	−3.249	<0.001	1.438
Participation in cultural activities	0.716	0.687	11.572	<0.001	1.438

*B*, unstandardized coefficient; β , standardized coefficient; VIF, variance inflation factor.

Based on the regression results presented in Table 4, the regression equation can be written using the unstandardized coefficients ( β ) as follows:

$$Y=1.971-0.304X_{1}+0.716X_{2}$$ (2)

where Y is the community cultural heritage preservation initiatives, $X_{1}$ is the cultural heritage preservation awareness, and $X_{2}$ is the participation in cultural activities. Based on Equation 2, community cultural heritage preservation initiatives increase as participation in cultural activities increases, holding other factors constant, indicating that active involvement plays a meaningful role in strengthening community-led preservation efforts. In contrast, awareness of cultural heritage preservation shows a negative association, suggesting that active cultural participation is a key factor in strengthening community cultural heritage preservation initiatives.

### 4.2 Qualitative results

The qualitative phase was conducted to explain three quantitative findings from the first phase. First, the survey results showed that heritage awareness was positively associated with participation in cultural activities. Second, awareness appeared to play a stronger role in explaining perceptions of LGU-led preservation initiatives. Third, participation emerged as a stronger predictor of community-led preservation initiatives, whereas awareness alone did not. The thematic analysis, therefore, focused on how participants described the movement from awareness to action, the role of local government in shaping preservation behavior, and the reasons community initiatives depended more strongly on active involvement than on awareness alone.

The qualitative results begin with a word cloud of the most frequent terms in the interview transcripts. This is followed by a table of key nodes, reference counts, and thematic mapping, after which the identified themes are briefly discussed using selected transcript excerpts.

#### 4.2.1 Thematic analysis

**Figure 5:**
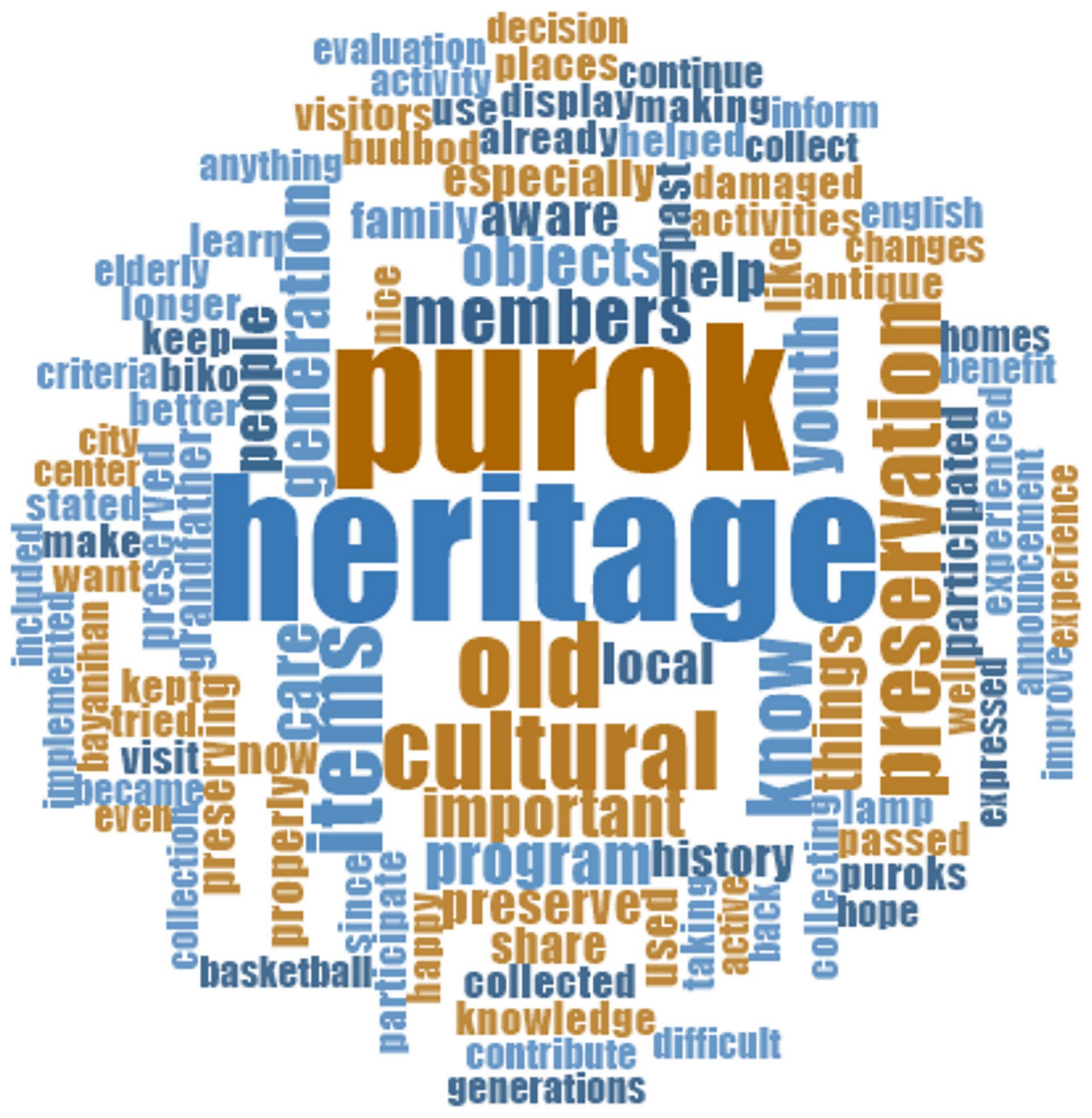
Word cloud showing dominant themes from the interview transcripts.

The word cloud in Figure 5 presents a summary of word frequencies generated in NVivo, serving as an initial step in node identification that informed the emerging themes within a concept-driven coding framework guided by the Uses of Heritage perspective (Smith, 2006). Initial open coding identified meaningful units from the transcripts, which were then organized using six guiding categories: heritage as a cultural process, heritage as experience, heritage as identity, the intangibility of heritage, memory and remembering, and heritage as performance. Codes were iteratively refined and clustered into broader themes grounded in the data. The results of these procedures are presented in Table 5 (Group 1: Actively Engaged Informants; Informants 1–10) and Table 6 (Group 2: Less-Involved Informants; Informants 11–20).

**Table 5:** Thematic results from focus group discussions with actively engaged informants.

Major theme	Excerpts	Codes	Category	Sub-theme
*Lived engagements in local heritage preservation*	“We can help by taking care of the heritage…” (Inf 3)	Active care of artifacts	Participation in preservation	Hands-on participation and care
	“We have learned to keep and value things…” (Inf 4)	Valuing heritage	Awareness development	Informed heritage engagement
	“I brought my lamp for display…” (Inf 5)	Contribution of artifacts	Community participation	
	“It’s difficult… but nice when collected.” (Inf 8)	Effort in preservation	Challenges in engagement	Challenges in heritage engagement
	“We take care of this lamp… part of our heritage.” (Inf 10)	Personal attachment	Family heritage value	Personal and familial connection
*Motivational pathways to sustained heritage preservation*	“To improve our purok.” (Inf 1)	Community improvement	Collective motivation	Community-oriented motivation
	“For the younger generation to see…” (Inf 3)	Future-oriented motivation	Intergenerational value	Intergenerational motivation
	“Need a safe place for display…” (Inf 4)	Need for facilities	Structural support	Need for institutional support
	“Mandated by LGU…” (Inf 6–8)	Policy-driven participation	Institutional influence	Compliance-driven participation
	“We just complied.” (Inf 9)	Passive compliance	External motivation	
	“To pass it to the next generation…” (Inf 10)	Legacy preservation	Personal motivation	Personal and cultural legacy
*Stewardship in heritage preservation*	“I will help preserve…” (Inf 1)	Willingness to contribute	Active involvement	Active stewardship
	“Encourage others to bring objects…” (Inf 2)	Community encouragement	Collective action	Community mobilization
	“We still do not know how to preserve…” (Inf 3)	Lack of knowledge	Skill gap	Need for capacity building
	“Responsibility is divided…” (Inf 4)	Shared responsibility	Family involvement	Shared responsibility
	“Share what we learned…” (Inf 6)	Knowledge sharing	Education	Knowledge transmission
	“Teach the next generation…” (Inf 10)	Intergenerational teaching	Continuity	Intergenerational stewardship

**Table 6:** Thematic results from focus group discussions with less-involved informants.

Major theme	Key phrase	Codes	Category	Sub-theme
*Limited awareness leading to non-participation in cultural heritage preservation*	“Not aware that a cultural heritage preservation program existed” (Inf 12)	Lack of awareness	Information gap	Limited awareness and non-participation
	“Did not know, but I’m willing to help” (Inf 13)	Willingness despite non-awareness	Conditional participation	Emerging interest in participation
	“Unable to participate because I was at work when they announced the activity…” (Inf 17)	Inaccessibility of activities	Situational barrier	Communication constraints and passive participation
	“Did not know…did not hear any announcement” (Inf 20)	Lack of information dissemination	Communication barrier	
*Cultural heritage preservation as a source of identity, continuity, and community meaning*	“Cultural heritage is the remnant of the past” (Inf 11)	Heritage as a connection to history	Historical consciousness	Heritage as identity and historical meaning
	“They made our place unique” (Inf 12)	Community uniqueness	Local identity	Heritage as community identity and pride
	“History and culture are important… help us understand the past” (Inf 14)	Understanding history	Historical learning	Heritage as a tool for education and knowledge transmission
	“Share the purok heritage with the youth” (Inf 16)	Intergenerational sharing	Knowledge transmission	Intergenerational responsibility and continuity
	“Carry on the legacy entrusted by the older generation” (Inf 17)	Legacy preservation	Future responsibility	
	“Connected to our roots” (Inf 19)	Connection to roots	Sense of belonging	Emotional and social meaning of heritage
*Youth marginalization and the need for inclusive, adaptive heritage governance*	“Have not experienced making a suggestion” (Inf 11)	Lack of youth voice	Exclusion from decision-making	Limited youth voice and participation in decision-making
	“Not confident enough to suggest anything” (Inf 13	Low confidence in expressing ideas	Participation constraint	
	“Coordinate the program with the youth or with all the purok members” (Inf 15)	Inclusive participation	Community collaboration	Call for inclusive and participatory approaches
	“Make an announcement if there is an activity” (Inf 16)	Need for better communication	Improved information dissemination	Need for improved communication
	“Teach the younger members so their knowledge remains with us” (Inf 18)	Knowledge transfer	Heritage education	Need for documentation and knowledge preservation
	“Use digital preservation to share heritage” (Inf 19)	Technological innovation	Adaptive governance	Desire for modernization and innovation
	“Be taught how to properly care/preserve/manage the old things” (Inf 20)	Need for training and guidance	Capacity building	Need for better heritage management

##### 4.2.1.1 Theme 1: lived engagements in local heritage preservation

The analysis reveals that actively engaged informants demonstrate heritage preservation through direct, practical involvement in safeguarding and valuing local artifacts and practices. Participants described concrete actions such as caring for heritage objects, contributing personal or family items for display, and recognizing the importance of preserving these materials as part of collective identity. This reflects heritage as a lived and embodied practice rather than a purely conceptual or institutional activity.

The theme also highlights the role of awareness development in shaping participation, where engagement leads to increased appreciation and valuation of heritage resources. However, participants also noted practical challenges in preservation activities, indicating that engagement is not without constraints. Lived engagement within the Purok system is a key mechanism for actively sustaining heritage within the community. Within this context, participation is embedded in everyday social interactions and collective practices that reinforce awareness, value, and care for local heritage resources.

##### 4.2.1.2 Theme 2: motivational pathways to sustained heritage preservation

Thematic analysis identified that sustained engagement in heritage preservation is driven by multiple and interacting motivational pathways. Participants expressed community-oriented motivations, such as improving the purok and preserving heritage for future generations. Intergenerational concerns were also evident, with informants emphasizing the importance of transmitting cultural materials and values to younger residents.

Motivation was likewise shaped by structural and institutional factors, including the need for adequate facilities and the influence of local government mandates, which sometimes resulted in compliance-driven participation. Alongside these external drivers, personal motivations such as legacy preservation also emerged. Within the Purok system, participation in heritage preservation is sustained through a combination of intrinsic, communal, and institutional motivators. These intersecting drivers shape how residents engage in preservation activities, reflecting the Purok’s role as a local structure that facilitates both individual commitment and collective action.

##### 4.2.1.3 Theme 3: stewardship in heritage preservation

Thematic analysis reveals that stewardship within the Purok system is characterized by shared responsibility, active community involvement, and efforts toward intergenerational continuity. Participants described preservation as a collective duty, with responsibilities distributed among family members and community actors, thereby reinforcing a sense of joint ownership of heritage resources.

At the same time, stewardship is shaped by capacity-related challenges, particularly limited knowledge and skills in proper preservation practices. Despite these constraints, participants emphasized initiatives such as knowledge sharing, community encouragement, and teaching younger generations. Within the Purok context, stewardship emerges as a socially embedded practice that supports both the maintenance and transmission of cultural heritage across generations.

##### 4.2.1.4 Theme 4: limited awareness leading to non-participation in cultural heritage preservation

The thematic analysis reveals that non-participation among less-involved informants is largely associated with limited awareness of cultural heritage preservation initiatives within the Purok system. Several participants reported they were not informed about the program’s existence or did not receive sufficient notice of scheduled activities, indicating gaps in communication and information dissemination at the community level.

In some cases, non-participation occurred despite expressed willingness to participate, suggesting that interest existed but was constrained by a lack of information. Additional barriers, such as work commitments and missed announcements, also contributed to reduced involvement. Within the Purok context, these findings highlight that participation in heritage preservation relies heavily on accessible communication channels and timely dissemination of information, which remain essential for inclusive engagement.

##### 4.2.1.5 Theme 5: cultural heritage preservation as a source of identity, continuity, and community meaning

The thematic analysis reveals that less-involved informants view cultural heritage preservation as closely linked to identity formation, historical understanding, and community meaning. Participants described heritage as a connection to the past that helps define local uniqueness and fosters a sense of pride in their community. These perspectives indicate that, even with limited direct participation, heritage is recognized as an important reference for collective identity.

The data also highlight the role of heritage in supporting continuity across generations. Informants emphasized the importance of transmitting knowledge and values to younger members to ensure the cultural legacy is maintained and understood over time. Within the Purok system, these views reflect how cultural heritage functions as a shared symbolic resource that reinforces belonging, strengthens awareness of roots, and supports continuity of community memory.

##### 4.2.1.6 Theme 6: youth marginalization and the need for inclusive, adaptive heritage governance

The thematic analysis reveals that youth participation in cultural heritage preservation within the Purok system remains limited due to restricted involvement in decision-making processes and low confidence in expressing ideas. Several participants reported not being given opportunities to contribute suggestions, while others hesitated to share their inputs, indicating barriers to active engagement among younger members.

At the same time, participants articulated the need for more inclusive and coordinated approaches that involve youth and other community members in program planning and implementation. Suggestions included improved communication through regular announcements, structured coordination with youth groups, and provision of training to strengthen heritage management skills. There was also interest in integrating digital tools for documentation and sharing of cultural heritage. Within the Purok context, these perspectives point to the need for more participatory and adaptive governance mechanisms that expand youth involvement and support sustainable heritage preservation practices.

### 4.3 Integration of quantitative and qualitative findings

The findings from the qualitative and quantitative analyses showed that members’ awareness significantly affects their participation in preserving cultural heritage. Studies have emphasized that understanding their cultural heritage can lead to active engagement in cultural activities and that increasing public awareness is vital for addressing potential concerns (Jones et al., 2019). Although raising awareness can be time-consuming and demand sustained commitment and local engagement (Liorančaitė-Šukienė and Jurėnienė, 2025), it remains one of the most effective approaches for fostering and maintaining respect for a community heritage. This means that government programs can influence members’ motivation to participate in preservation activities. Hence, providing policymakers with insights to go beyond ordinances. Government initiatives, such as introducing policies, providing education, and allocating funds for preservation, have increased community awareness of the importance of safeguarding heritage (Falkovskyi and Tarasov, 2025). It is further emphasized that participatory decision-making processes and community-based approaches in conservation governance are important in supporting the custodians of traditional knowledge (Maliao and Tóthmérész, 2026). This points to the importance of empowering communities to manage and preserve their cultural heritage effectively.

The findings present the concepts of duty and communal initiative, demonstrate the impact of local efforts on heritage preservation, and provide the stakeholders with a benchmark for enhancing the program. The willingness of members to participate in decision-making processes for heritage preservation (Chauhan, 2022) instills a sense of responsibility and connection to safeguarding cultural heritage (Agoes and Safari, 2024). This indicates that the members’ drive and vision in preserving cultural heritage for the future generation to appreciate and value their local heritage. They are regarded as powerful learning tools and effective ways to explore local culture and history (Pedroso et al., 2023). Interacting with their heritage provides them with details and information essential for understanding local culture and history (Not et al., 2019). This emphasizes their role in motivating members to preserve their cultural heritage. The program develops a deeper understanding of their local history and its connection to national heritage, strengthening their sense of ownership and responsibility for heritage protection (Kintanar and Barretto-Tesoro, 2020). The members acknowledged their role in safeguarding heritage assets and are willing to engage in conservation programs that raise awareness and support sustainable preservation.

The qualitative findings indicate that participation in cultural activities is the strongest predictor of both LGU and community cultural heritage preservation initiatives, while cultural heritage awareness shows a weaker, and in some cases non-significant, effect. This pattern is consistent with qualitative accounts from the less engaged group, in which non-participation was primarily linked to limited awareness, insufficient information dissemination, and situational constraints such as work-related absences. These findings align with recent literature, which indicates that community participation in living heritage conservation is strongly influenced by awareness, access to information, and community-based education mechanisms (Abdul Aziz et al., 2023). This means that awareness alone does not automatically translate into active participation, as sustained engagement requires enabling structures such as effective communication channels and participatory initiatives.

In addition, although respondents recognized cultural heritage as a source of identity, continuity, and community meaning, this recognition did not consistently translate into active participation, reflecting a persistent gap between cultural appreciation and preservation behavior. This is consistent with prior research indicating that awareness and symbolic value alone are insufficient to sustain collective action without experiential engagement and inclusive governance structures (Smith, 2006). The stronger influence of participation compared to awareness further supports the view that heritage preservation is reinforced through active involvement rather than cognitive recognition alone. The expressed need for inclusive coordination, improved communication, and youth engagement aligns with the literature, which emphasizes that adaptive and participatory governance is essential for sustaining community-based heritage initiatives (Banda et al., 2024). Such governance structures strengthen community ownership and support the long-term preservation of cultural heritage.

## 5 Discussion

This section integrates quantitative and qualitative results to provide a comprehensive understanding of cultural heritage preservation. Quantitative data show the patterns and strengths of relationships among awareness, participation, and preservation initiatives, while qualitative data offer thematic explanations that help interpret these patterns. The combined insights are discussed in three parts: (1) awareness, involvement, and preservation practices, (2) insights into community heritage preservation, and (3) the *Purok* as a center for community action.

### 5.1 Awareness, involvement, and preservation practices

The convergence of quantitative and qualitative findings indicates that residents generally demonstrate clear awareness of cultural heritage preservation, which is closely associated with their involvement in heritage-related activities. However, participants who identified as less involved or dissenting showed comparatively limited awareness of existing heritage programs, which contributed to their lower levels of participation. This reflects a broader pattern in heritage research, in which educated and engaged communities show a more substantial commitment to sustaining cultural values and practices (Pai et al., 2025). This aligns with recent findings indicating that community participation models strengthen community identity (Abdul Aziz et al., 2023) and that local awareness of cultural heritage resources serves as a key determinant of engagement in heritage-related activities (Mteti et al., 2025). The findings may be linked to active public engagement in heritage initiatives, with residents in Liloan, Cebu, participating in local festivals, such as the *Rosquillos* Festival, and heritage activities at Bagacay Point Lighthouse, also known as “*Parola*.” Such engagement strengthens appreciation and long-term stewardship of cultural assets, moving preservation beyond awareness toward sustained practice.

Qualitative insights such as “lived engagements,” “motivational pathways,” and “youth marginalization” reinforce the idea that community members interpret awareness through participation and shared cultural narratives, rather than solely through knowledge. Participants noted that having a visible, organized storage area for heritage items encouraged them—especially the youth—to actively preserve and learn about the history and use of each item, even when guidance was limited. However, findings also suggest that youth marginalization persists in other aspects of heritage governance, where limited inclusion in decision-making and program communication continues to constrain sustained participation. This aligns with calls for more participatory approaches in heritage management (Del Baldo and Demartini, 2021). These lived experiences mirror scholarly discussions about how community narratives and shared activities deepen connection to heritage and reinforce responsibility toward its care. For example, community-centric approaches to heritage preservation emphasize local ownership and involvement as essential for sustainability (Çardak, 2025). The quantitative and qualitative findings suggest that awareness in this Philippine context is dynamic, formed by community involvement, and that heritage practices thrive where collective action comes together.

### 5.2 The Purok as a center for community action

The findings from both quantitative and qualitative strands point to the *Purok*, a small neighborhood cluster within a *Barangay* (Matthies, 2017), as an effective platform for mobilizing localized community action in the Philippines. Although general awareness did not directly predict community initiatives, higher participation in activities was associated with stronger community-led efforts, suggesting the *Purok* functions as an organizing unit that channels involvement into concrete action. Qualitative themes of steering heritage practices and motivational pathways to sustained preservation reflect how the *Purok* units serve as social hubs that translate shared values into collective initiatives. This reinforces findings from disaster risk management research, which show that *Purok* systems enhanced community collaboration and responsiveness through established local networks and coordinated engagement (De Leon, 2021). This implies that localized structures amplify volunteerism and collective agency, enabling residents to work together on heritage preservation beyond individual participation alone.

Moreover, the role of the *Purok* units as community action centers aligns with broader evidence on community-based organizational frameworks that strengthen socio-cultural initiatives through trust and shared responsibilities. Global research on participatory heritage engagement shows that community involvement in cultural heritage conservation fosters collaborative decision-making and sustained stewardship beyond top-down approaches. For example, the interviews revealed that engagement in heritage preservation often depends on personal priorities, with the current generation, particularly younger residents, showing less interest as they are more focused on digital activities and online interactions. This contrasts with studies suggesting that Generation Z shows strong intentions to participate in heritage activities (Wang et al., 2025), indicating that sustaining intergenerational and participatory engagement may require targeted strategies to motivate younger community members. Such models emphasize community cooperation and collective agency in heritage work—a pattern reflected in this study where participation within community clusters (e.g., *Purok* units) translated awareness into sustained preservation practices.

Another case highlights a dynamic sense of place that extends beyond geographically bounded communities, where *glocal* communities formed through social ties, emotional connections, and digital platforms support sustained people–place bonding in contemporary heritage conservation (Chen and Wang, 2024). Consistent with this, the expressed need for inclusive coordination, improved communication, and youth engagement within local heritage systems underscores the importance of adaptive and participatory governance in sustaining community-based heritage initiatives, thereby strengthening community ownership and long-term preservation outcomes (Banda et al., 2024). These studies reinforce the idea that organized community units provide essential structures that convert motivation and awareness into long-term socio-cultural engagement and that efforts should focus on helping younger generations understand and participate in heritage preservation.

### 5.3 Insights into community heritage preservation

From a policy perspective, the findings point to the need for Philippine cultural heritage programs to institutionalize community-based approaches that recognize local action units as active partners in heritage preservation. National and regional policies may benefit from moving beyond awareness campaigns toward supporting sustained participation, capacity building, and resource allocation at the community level. Our findings have implications for local governments and policymakers to strengthen public participation in heritage conservation, such as the preservation of green cultural heritage (Xie et al., 2024), while also pointing to new theoretical directions for studying community engagement. Such an approach aligns with ongoing decentralization efforts in the Philippines and supports inclusive governance in cultural heritage management.

For practitioners, the findings reinforce the relevance of participatory and community-based theories, particularly those emphasizing collective agency and social learning. Frameworks such as cultural capital theory help explain how trust, shared responsibilities, and repeated interaction enable communities to sustain heritage practices over time (Bourdieu, 2018). Community members may be highly aware of cultural heritage preservation but remain inactive due to limited involvement in decision-making, a lack of opportunities, and an over-reliance on leadership. Awareness becomes effective when embedded in social structures that encourage collaboration and mutual accountability. This suggests that successful preservation initiatives depend not only on technical expertise but also on fostering relationships, facilitating participation, and supporting community networks, with digitization and social innovation playing a key role in enabling rural cultural heritage to be actively practiced and transmitted across generations (García-Mieres et al., 2025). This was reflected in the study, as regression results and interview transcripts showed that greater participation and engagement were associated with more sustained heritage practices.

## 6 Conclusion

Despite growing interest in community-based heritage preservation, few studies have explored how localized community clusters sustain cultural heritage preservation initiatives. To address this gap, this study employed a sequential explanatory mixed-methods design that combined quantitative surveys and qualitative interviews. A total of 264 members from six selected Purok units in the Municipality of Liloan, Philippines, participated in the survey using a multi-stage sampling approach. For the qualitative component, 10 informants were purposively selected based on their level of engagement in cultural heritage activities, with a further 10 added to capture less-involved perspectives, for a total of 20. The convergent analysis of survey and interview data revealed several key findings, along with practical and theoretical insights for policy and heritage preservation practice. From these results, three salient findings emerged that highlight the patterns and dynamics of community-based heritage preservation.

First, awareness of cultural heritage was positively associated with participation in heritage-related activities, indicating that greater knowledge of heritage is associated with greater engagement. However, awareness alone is not sufficient to drive active involvement. Interviews revealed that community programs actively encouraged members to preserve local heritage items and traditions. Second, community clusters, or *Purok* units, serve as effective centers for coordinated action, translating individual willingness into collective preservation efforts. This is supported by the regression results, which showed that participation in cultural activities significantly predicted community initiatives, indicating that individual engagement drives broader, organized preservation efforts. Interviews reinforced this, with participants describing how working together encouraged coordinated activities, such as collecting heritage items and sustaining local delicacies. Third, organized community units help translate motivation and awareness into sustained heritage engagement by reinforcing collective identity, continuity, and shared meaning. This was reflected in the thematic analysis, which identified collective activities and stewardship practices that reinforce community participation and strengthen continuity of shared identity and meaning. However, greater attention is needed to encourage less-involved youth to understand and actively engage in preservation activities to sustain these processes over time.

These findings emphasize the importance of community-based approaches in cultural heritage preservation. Policies and programs should support sustained participation, capacity building, and local engagement, guided by theoretically framed directions that link community action, social learning, and collective responsibility. Effective preservation relies not only on technical expertise but also on fostering relationships, collaboration, and collective action. *Purok* units serve as potential savers of heritage items handed down through generations, and younger generations must be empowered, as their digital skills can support innovative ways to practice and transmit heritage. Greater participation and engagement, as shown in the quantitative and qualitative results, reinforce long-term preservation outcomes and offer guidance for both policy and future research.

Like several related studies, this research has limitations that should be considered. The study was conducted in a single municipality, which limits the transferability of the findings to other local contexts, and participant recruitment relied on nominations from Purok presidents, with the qualitative participants selected from the same survey pool. The quantitative sample remained bounded and community-specific, so the findings should not be interpreted as broadly generalizable beyond the case site. Future research could examine multiple municipalities, compare urban and rural settings, or explore specific digital and social innovation interventions in heritage preservation. In particular, future studies should focus on younger generations, investigating their digital skills and uncovering associations among latent factors to understand better how they can be trained and empowered to actively participate in heritage preservation.

## Data Availability

The raw data supporting the conclusions of this article will be made available by the authors, without undue reservation.
